# Concurrence of Neurocysticercosis and Hemangioblastoma

**DOI:** 10.4269/ajtmh.24-0457

**Published:** 2024-10-01

**Authors:** Oscar H. Del Brutto, Juan Carlos Garcés, Denisse A. Rumbea, Iván X. Mena

**Affiliations:** ^1^ School of Medicine and Research Center, Universidad Espíritu Santo – Ecuador, Samborondón, Ecuador;; ^2^ Laboratorio de Patología, Patología Quirúrgica y Citopatología, Hospital-Clínica Kennedy, Guayaquil, Ecuador;; ^3^ Department of Neurosurgery, INTERHOSPITAL S.A., Guayaquil, Ecuador

**Figure 1.**
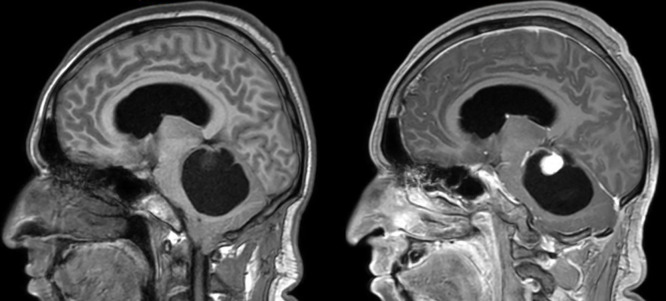
T1-weighted magnetic resonance imaging in the sagittal plane before (left) and after (right) contrast medium administration showing a cystic lesion with an enhancing mural nodule in the cerebellum.

**Figure 2.**
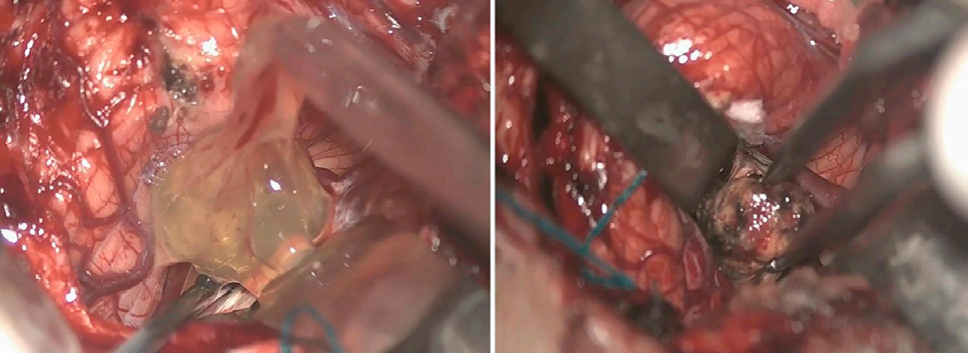
Trans-operative findings. Several yellowish membranes highly suggestive of racemose cysticerci are observed in the subarachnoid space of the posterior fossa (left), and a solid nodule is visualized in the upper part of the cerebellar vermis (right).

**Figure 3.**
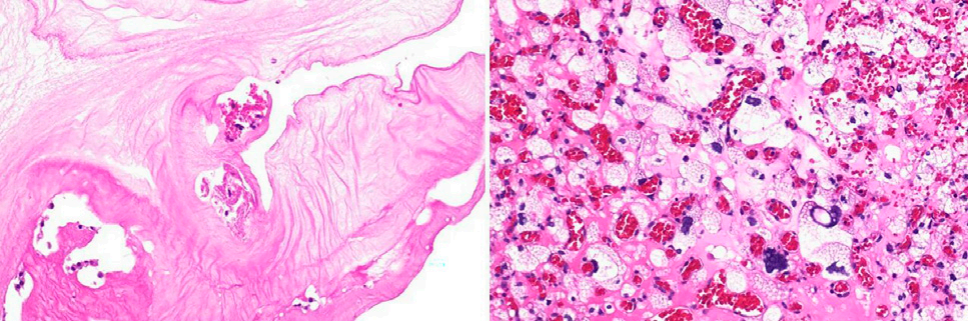
Histopathologic specimens (hematoxylin and eosin stain) showing the membranes forming the cystic component of the lesion, consistent with cestode membranes (left) and a highly vascularized tumoral nodule characterized by stromal cells containing foamy vacuolated cytoplasm (right), typical of hemangioblastoma.

A previously healthy 29-year-old man was admitted because of progressive headache, dizziness, uncoordinated movements in the right side of the body, and trouble walking during the past 8 months. Fundoscopic examination was normal. Brain magnetic resonance imaging showed obstructive hydrocephalus together with a cystic space–occupying lesion in the posterior fossa; after contrast medium administration, a tumoral nodule was noted in the upper part of the cyst (Figure 1). As a first procedure, a ventricular shunt was placed for relief of hydrocephalus. Then, the patient underwent a suboccipital craniectomy for resection of the cerebellar lesion. Trans-operative findings included the presence of yellowish membranes suggestive of racemose cysticerci that surrounded a solid tumoral nodule of about 15 mm in diameter located in the upper cerebellar vermis (Figure 2). Of interest, these membranes formed the total cystic component of the lesion, which did not have an additional cystic component. Cysticercal membranes and the nodule were successfully removed. Histopathologic examination showed typical cestode membranes that comprised the total cystic component of the lesion and a solid nodule consistent with an hemangioblastoma (Figure 3).

Hemangioblastomas are benign tumors that may occur sporadically or in the context of von Hippel-Lindau disease, a genetic disorder that is associated with tumors in different organs, in particular the central nervous system (CNS).^1^ Although pathogenesis of hemangioblastoma is not totally understood, it has been postulated that these highly vascularized tumors grow under conditions of increased angiogenesis.^2^

Cysticercosis has been associated with glial tumors.^3^ Pathogenetic mechanisms explaining the oncogenic effects of cysticerci include inflammation, release of nitric oxide, inhibition of tumor suppression genes, loss of regulatory mechanisms involved in the immunological surveillance against cancer, and transfer of genetic material from the parasite to the host, resulting in malignant transformation of astrocytes.^4^ Although these proposed mechanisms explain the association between cysticercosis and certain cerebral neoplasms, they do not account for the association with hemangioblastoma.

The association between cysticerci and overexpressed angiogenesis in a rat model of neurocysticercosis has also been demonstrated.^5^ According to that study, abnormal angiogenesis was predominant in the vicinity of parasites, suggesting a local effect. In the present case, it is possible that a slow-growing racemose cysticercus favored the development of the solid component of the tumor. If confirmed in further reports in humans, our findings would support a potential causal relationship between neurocysticercosis and vascular tumors of the CNS, explaining this hitherto unrecognized complication of this parasitic disease.
